# Supplementary material for: Temperature relaxation in strongly-coupled binary ionic mixtures
Source: Nat Commun. 2022 Jan 10;13:15. doi: 10.1038/s41467-021-27696-5 (PMC8748956; doi:10.1038/s41467-021-27696-5)
Supplement: Supplementary file 1 — Supplementary Information [file 41467_2021_27696_MOESM1_ESM.pdf]

Supplementary Information for "Temperature relaxation in strongly-coupled binary ionic mixtures"

R. Tucker Sprenkle, L. G. Silvestri, M. S. Murillo, S. D. Bergeson

Supplementary Note 1

In plasma physics the temperature is often defined pragmatically as

$$k_B T_\alpha(t) = \frac{m_\alpha}{3N_\alpha} \sum_{i=1}^{N_\alpha} v_i^2(t), \tag{1}$$

This arises from a discretization of the distribution function

$$k_\mathrm{B} T(t) = \frac{m}{3} \int d^3v \, (\mathbf{v} - \langle \mathbf{v} \rangle)^2 f(\mathbf{v}, t), \tag{2}$$

where $f(\mathbf{v}, t)$ is the microscopic form of the velocity distribution

$$f(\mathbf{v}, t) = \sum_{i=1}^{N} \delta(\mathbf{v} - \mathbf{v}_i(t)). \tag{3}$$

In homogeneous plasmas there is no drift, $\mathbf{u} = \langle \mathbf{v} \rangle = 0$ and Eq. (1) is recovered. The temperature could be defined alternatively by fitting the binned one-dimensional velocity distribution to a Maxwellian,

$$\mathcal{M}\left(\frac{v}{\sigma}\right) = \frac{1}{\sigma\sqrt{2\pi}} \exp\left(-\frac{v^2}{2\sigma^2}\right), \tag{4}$$

with σ as the fit parameter. The temperature would then be $k_\mathrm{B} T = m\sigma^2$. As the system approaches equilibrium, these two definitions of temperature will agree. The extent to which they do not agree is a measure of the non-equilibrium nature of the system.

Quantifying the departure of the temperature defined using Eq. (1) from the equilibrium value can be done using a Hermite polynomial expansion (*1,2*). For a normalized one-dimensional velocity distribution $f(v, t)$ the expansion is

$$f(v, t) = \mathcal{M}\left(\frac{v}{\sigma}\right) \sum_{n=0} \frac{1}{n!} a_n(t) \, \mathcal{H}_n\left(\frac{v}{\sigma}\right), \tag{5}$$

where $\mathcal{H}_n(v/\sigma)$ are the probabilistic Hermite polynomials of order n and the Maxwellian distribution acts as a weight function. The Hermite coefficients are found using orthogonality relations.

$$\int f(v) \mathcal{H}_m\left(\frac{v}{\sigma}\right) dv = \int \mathcal{M}\left(\frac{v}{\sigma}\right) \sum_{n=0} \frac{1}{n!} a_n \, \mathcal{H}_n\left(\frac{v}{\sigma}\right) \mathcal{H}_m\left(\frac{v}{\sigma}\right) = a_m, \tag{6}$$

where the time dependence has been suppressed for brevity.

Critical to Hermite analysis is a correct value for the width of the Maxwellian weight function σ. When the distribution function $f(v,t)$ is itself a Maxwellian distribution with rms width equal to σ, the Hermite coefficients a_n will all vanish except for $a_0 = 1$. However, if $f(v,t)$ is Maxwellian with a width different from σ, the expansion in Eq. 5 systematically gives non-zero values for the a_n coefficients.

When the value of σ is not known beforehand, it can be estimated using the second moment of the velocity distribution,

$$\langle v^2(t) \rangle = \int dv \; v^2 f(v,t), \tag{7}$$

While this is a logical choice, it complicates the Hermite analysis because σ is a parameter in the Hermite polynomials. In particular, the second moment can be rewritten as,

$$\langle v^2(t) \rangle = \int dv \; \left[\mathcal{H}_0(v) + \mathcal{H}_2(v)\right] f(v,t), \tag{8}$$

leading to

$$k_{\rm B}T(t) = m\langle v^2(t)\rangle = m\sigma^2\left[1 + a_2(t)\right]. \tag{9}$$

The width of the weight function σ and the Hermite coefficient a_2 are coupled together.

Just as $\langle v^2\rangle/\sigma^2 = 1 + a_2$, higher moments of the velocity distribution are also linear combinations of the Hermite coefficients, *e.g.* $\langle v^4\rangle/\sigma^2 = 3a_0 + 6a_2 + a_4$. Without an unambiguous determination of σ, the Hermite coefficients cannot be determined from the velocity moment calculations alone.

It might be assumed that the values of σ and a_n could be extracted directly from a fit of $f(v,t)$ to Eq. (5). This is reliable only when the noise distribution in each velocity bin of $f(v,t)$ is Gaussian with zero mean. However, the MD distributions are necessarily non-negative. Towards the wings of the distribution, where there are relatively few particles per bin, the noise distribution in each bin becomes binomial and has a non-zero mean. This feature causes stochastic changes in the determination of σ and a_2 because of the coupling relationship in Eq. (9). It also artificially biases the determination of a_n with $n \geq 4$. It might be possible to artificially augment the MD noise distribution in each velocity bin in an attempt to force the condition of zero mean. However, the reliability of this approach is not known. This remains an area of future research.

As discussed in the main text, ratios of the velocity moments give insight into non-Maxwellian departures of the distribution. In the case of a perfect Maxwellian distribution moments higher than $n = 2$ satisfy the relation

$$\langle v^n \rangle = \begin{cases} 0 & \text{if } n \, \text{odd}, \\ (\langle v^2\rangle)^{n/2}(n-1)!! & \text{if } n \, \text{even}. \end{cases} \tag{10}$$

Therefore the moment ratios $\left\langle v^4\right\rangle/3\left\langle v^2\right\rangle^2$, $\left\langle v^6\right\rangle/15\left\langle v^2\right\rangle^3$, and $\left\langle v^8\right\rangle/105\left\langle v^2\right\rangle^4$ are all equal to unity.

Calculations of moment ratios and Hermite coefficients are influenced by the small numbers of particles in the wings of the MD distribution at large velocities. The values of the coefficients and moment ratios therefore depend on how far out into the wings the analysis persists. The moment ratios included in the manuscript span the distribution out to $\pm 7\sigma$, where σ is estimated using a Gaussian fit to the MD distribution.

Supplementary References

1. R. L. Liboff, *Kinetic theory: classical, quantum, and relativistic descriptions* (Springer Science & Business Media, 2003).

2. H. Grad, On the kinetic theory of rarefied gases. *Communications on Pure and Applied Mathematics* **2**, 331-407 (1949).
